# Predictive physiological anticipatory activity preceding seemingly unpredictable stimuli: An update of Mossbridge *et al*’s meta-analysis

**DOI:** 10.12688/f1000research.14330.2

**Published:** 2018-07-17

**Authors:** Michael Duggan, Patrizio Tressoldi

**Affiliations:** 1 Independent researcher, Birmingham, UK; 2 Dipartimento di Psicologia Generale, Universita di Padova, Padova, Italy

**Keywords:** pre-stimulus activity, anticipatory physiology, temporal processing, psychophysiology, presentiment

## Abstract

**Background:** This is an update of the Mossbridge *et al*’s meta-analysis related to the physiological anticipation preceding seemingly unpredictable stimuli which overall effect size was 0.21; 95% Confidence Intervals: 0.13 - 0.29

**Methods:** Nineteen new peer and non-peer reviewed studies completed from January 2008 to June 2018 were retrieved describing a total of 27 experiments and 36 associated effect sizes.

**Results:** The overall weighted effect size, estimated with a frequentist multilevel random model, was: 0.28; 95% Confidence Intervals: 0.18-0.38; the overall weighted effect size, estimated with a multilevel Bayesian model, was: 0.28; 95% Credible Intervals: 0.18-0.38.

The weighted mean estimate of the effect size of peer reviewed studies was higher than that of non-peer reviewed studies, but with overlapped confidence intervals: Peer reviewed: 0.36; 95% Confidence Intervals: 0.26-0.47; Non-Peer reviewed: 0.22; 95% Confidence Intervals: 0.05-0.39.

Similarly, the weighted mean estimate of the effect size of Preregistered studies was higher than that of Non-Preregistered studies: Preregistered: 0.31; 95% Confidence Intervals: 0.18-0.45; No-Preregistered: 0.24; 95% Confidence Intervals: 0.08-0.41.

The statistical estimation of the publication bias by using the Copas selection model suggest that the main findings are not contaminated by publication bias.

**Conclusions:** In summary, with this update, the main findings reported in Mossbridge *et al*’s meta-analysis, are confirmed.

## Introduction

The human ability to predict future events has been crucial in our evolutionary development and proliferation over epochs of time, both from a species perspective, but also, on an individual level. Our day-to-day survival is predicated on a successful marriage of experience (e.g., memory) and sensory processing (e.g., perceptual cues); for example, on a very humid heavily overcast night, our perceptions and memories inform us that a thunder storm is possible and it might be intelligent to find shelter. Such behaviour is highly adaptive as it fosters survival based strategies and is perfectly explicable in terms of current theories of biological causality. Now imagine if such prognosticating ability was possible without any sensory or other inferential cues (see Mossbridge & Radin, 2018 for a review). Such seemingly inexplicable ability would definitely hold survival advantage, if they existed. For millennia people have been reporting strange feelings of foreboding that later transpired to have significance. Over the last 36 years these phenomena have been scrutinized in the laboratory in which a subject’s physiology is monitored before a randomly presented stimulus that is designed to evoke a significant post-stimulus response. Disturbingly, moments before the stimulus is presented there are physiological changes ahead of time. This effect is termed presentiment, or more recently, Predictive Anticipatory Activity ( Mossbridge *et al*., 2014). By 2012 a good number of these studies had been completed and it was deemed worthwhile to conduct a meta-analysis of the extant literature at the time. Mossbridge, Tressoldi and Utts located 42 studies published from 1978 to 2010, testing the presentiment hypothesis, out of which 26 enabled a true comparison between pre and post-stimulus epochs ( Mossbridge *et al*., 2012), that is the pre-stimulus physiological responses mirrored even if to a lesser degree, the post-stimulus responses.

Here two paradigms were used: either a randomly ordered presentation of arousing vs. neutral stimuli or guessing tasks in which the stimulus is the feedback about the participant’s guess (correct vs. incorrect). In both of these approaches it is difficult to envision mundane strategies that might explain the anomalous pre-stimulus effects observed, and indeed, Mossbridge *et al*., went to significant lengths in refuting the leading candidate – expectancy effects, both in the 2012 meta-analysis and in post-review exchanges with sceptical psychologists and physiologists. Regardless of the paradigm, a broad range of physiological measures were employed from skin conductance, heart rate, blood volume, respiration, electroencephalographic (EEG) activity, pupil dilation, blink rate, and/or blood oxygenation level dependent (BOLD) responses. These are recorded throughout the session, with a pre-determined anticipatory period of between 4 to 10 seconds, in which the any pre-stimulus effect is captured. The presentiment hypothesis calls for a difference between the pre-stimulus responses of the two stimulus categories and this is calculated across sessions. Mossbridge *et al*. found substantive evidence in favour of a presentiment effect concatenated to over 6 sigma – extreme statistical significance. Additionally, they also found evidence of presentiment effects from mainstream research programs ( Bierman, 2000) something that is becoming increasingly important as these effects become more widely known.

Because of the high profile nature of Mossbridge *et al*., (over 93,000 views as of January 2018) there has been a good number of replications in the few years since publication. We located an additional 26 studies describing 34 effect sizes from a dozen laboratories. The most striking aspect of this fresh database is the sheer variation in experimental approaches as researchers seek to tackle more process-oriented questions rather than continuing the proof-oriented work found in the earlier meta-analysis. Because expectancy effects have been proposed as a potential mechanism to explain at least some of the presentiment effect, it is noteworthy that several experiments in this fresh cohort of studies tackle this head on by only analysing the first trial of a run. These single-trial presentiment studies are expectancy free and are becoming more dominant in this research domain. Another interesting question that is probed in these new studies is the idea of utilizing pre-stimulus physiological activity to predict future events. This provides another objective measure of the validity of the presentiment effect. There are several studies that utilize this approach and they are discussed later on. Also of note we found several PhD theses describing presentiment research and a greater geographical spread than in 2012, both evidence of the increasing attention such research is garnering. Lastly, we found increasing dialogue between presentiment researchers and physicists interested in retrocausality – the idea that effects can precede their cause. This is witnessed in the recent AAAS retrocausality symposium in which several researchers participated and in which some of those papers made their way into this meta-analysis ( Sheehan, 2017).

## Methods

The whole procedure followed both the APA Meta-Analysis Reporting Standards ( APA Publications and Communications Board Working Group on Journal Article Reporting Standards, 2008), the Preferred Reporting Items for Systematic reviews and Meta-Analyses for Protocols (PRISMA) 2015 ( Moher *et al*., 2015) and the reporting standards for literature searches and report inclusion ( Atkinson *et al*., 2015). A completed PRISMA checklist can be found in Supplementary File 1.

### Study eligibility criteria

Study inclusion criteria were the analysis of both psychophysiological or neurophysiological signals before the random presentation of whichever type of stimulus, e.g. pictures, sounds etc. Randomization could be performed by using pseudo-random algorithms e.g. like those implemented in MatLab or E-Prime® or true random sources of random digits, e.g. TrueRNG.

It is important to point out that these eligibility criteria are different from those used by Mossbridge *et al.* Those authors selected only studies where the anticipatory signals mirrored the post-stimulus ones. In addition we included all studies that used anticipatory signals to predict future events independently of the presence of post-stimulus physiological signals. For example, some authors, e.g. Mossbridge (2015) used heart rate variability to predict winning i.e. $4, versus losing outcomes without recording the post-stimulus physiological activity associated with hits and misses. Our inclusion criteria are consequently more comprehensive than those used by Mossbridge *et al*., 2012

### Studies retrieval procedure

Both co-authors who are experts in this type of investigations, searched for studies through Google Scholar and PubMed by using the keywords: “presentiment” OR “anticipation” OR “precognition”. Furthermore, we emailed a request of the data of completed studies to all authors we knew were involved in this type of research. Even if Mossbridge *et al.* included all studies available up to 2010, we also searched studies that could have been missed in that meta-analysis. We searched all completed studies, both peer reviewed and non-peer reviewed, e.g. Ph.D dissertations, from January 2008 to June 2018.

### Study selection

**Figure 1.**
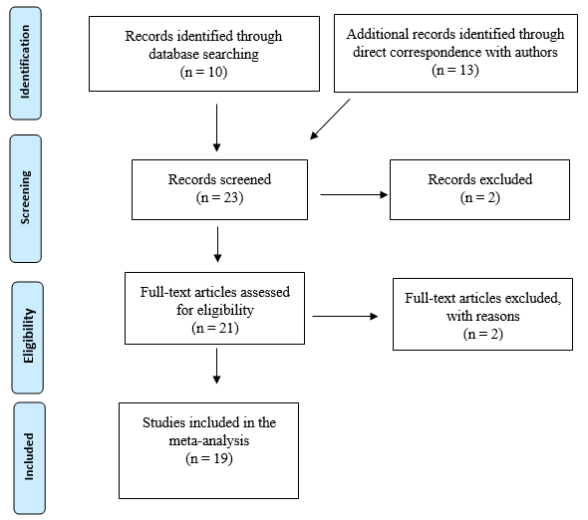
Flow-diagram of study selection.

Study selection is illustrated in the flow-diagram presented in Figure 1

Excluded records were studies where the psychophysiological variables were analysed only after and not before the stimuli presentations ( Jin *et al.*, 2013) and with an unusual procedure ( Tressoldi *et al.*, 2015), i.e. using heart rate feedback to inform a voluntary decision to predict random positive or negative events.

Records excluded after the screening were studies where authors did not agree to share their data for different reasons ( Baumgart *et al*., 2017; Modestino *et al*., 2011). Excluded studies revealed either statistically significant or trending evidence in support of the anticipation effect in most cases, thus reducing the concerns surrounding biased removal.

The references of the included studies are reported in Supplementary File 2.

### Coding procedure

The two co-authors agreed on the following coding variables: Authors; year of publication; participant selection: yes = selected according to specific criteria; no = selected without specific criteria; number of participants; number of trials; stimuli type; type of randomisation: pseudo or true random; psychophysiological signals, e.g. EEG, Heart Rate, etc.; anticipatory period; type of statistics; value of statistics and independently extracted them from the eligible studies. After the comparison, they discussed how to solve the inter-coder’ differences.

On the database we have added a note for each effect size, describing where we extracted the corresponding statistics in the original papers. The database along with all 19 papers are available from Tressoldi (2017). A summary of the selected studies along with their corresponding effect sizes, variance and standard error, is reported on Table S1 in the Supplementary File 3.

### Moderator variables

Apart from the overall effect, we chose to compare the following moderator variables, peer review (PeerRev, yes vs no) as a control of study quality. Given the low number of studies no further moderator analyses were carried out.

### Statistical methods

The standardized effect size *d* of each dependent variable, was estimated from the descriptive statistics (means, standard deviation and number of participants) when available. In all other cases, it was estimated by using the available summary statistics, i.e. paired t-test; Stouffer’s Z; etc. by using Lakens’ software ( Lakens, 2013) and the function *escalc ()* of the R package metaphor ( Viechtbauer, 2017).

All effect sizes were then converted into the Hedges’ *g* and the corresponding variance by using the formulae suggested by Borenstein *et al*. (2009) estimating an average correlation of 0.5 between the dependent variables.

Given our choice of keeping (not averaging) all effect sizes when multiple dependent variables were analysed, we estimated the overall random model weighted effect size by using the robumeta package ( Fischer *et al*., 2017) which implement a Robust Variance Estimation method when there are dependent effect sizes ( Tanner-Smith & Tipton, 2014).

In order to control the reliability of the results, a second analysis was carried out by using a multilevel approach as suggested by Assink & Wibbelink (2016) implemented with the metafor package ( Viechtbauer, 2010) and reported in the Table S2 in the Supplementary File 3.

A Bayesian meta-analysis was implemented with the brms package ( Bürkner, 2017).

A copy of the syntax is available here: https://doi.org/10.6084/m9.figshare.5661070.v1 ( Tressoldi, 2017)

## Results

### Descriptive statistics

Studies: Peer reviewed papers: 9; Non -Peer reviewed papers:10. Number of experiments: 27 contributed by 14 authors. Number of effect sizes: 36. Average number of participants: 97.5. Average anticipatory period: 3.5 seconds. Four studies were preregistered (see database).

The group analyses for males and females reported in three papers ( Mossbridge, 2014; Mossbridge, 2015; Singh, 2009), were considered independent effect sizes.

### Frequentist multilevel random model

**Figure 2.**
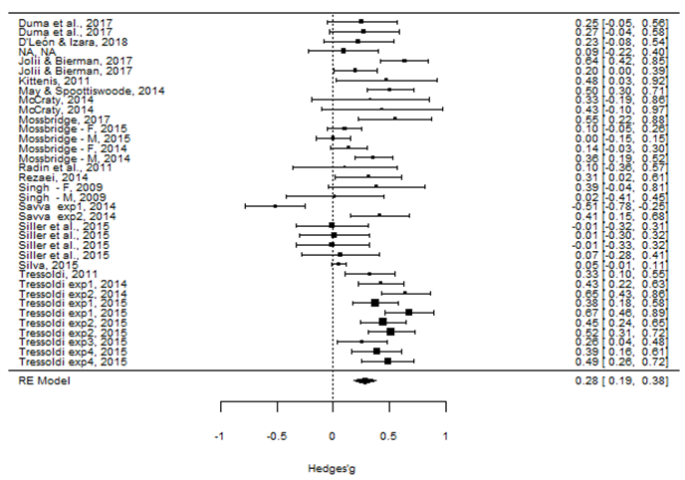
Forest plot of the frequentist multilevel random model analysis.

The forest plot is presented in Figure 2. The summary of the frequentist multilevel random model analysis is presented in Table 1 compared with the results obtained by Mossbridge *et al*., whereas the summary of the Bayesian multilevel random model meta-analysis is presented in Table 2.

**Table 1.** Results of the frequentist multilevel random model analysis.

	n	ES	95% Conf. Int.	*p*	I ^2^	τ ^2^
**Mossbridge** ***et al.***	**26**	**0.21**	**0.13 – 0.29**	**5.7×10 ^-8^**	**27.4**	**0.012**
**Overall**	**27**	**0.28**	**0.18 – 0.38**	**5.6×10 ^-6^**	**81.9**	**0.048**
Peer Reviewed	13	0.36	0.26 – 0.47	1×10 ^-14^	44.9	0.014
Non Peer Reviewed	14	0.22	0.05 – 0.39	0.014	85.2	0.048

n= number of experiments; ES= estimated effect size with corresponding 95% confidence intervals, *p* values; I ^2^: effect sizes heterogeneity; τ ^2^: effect size variance heterogeneity.

**Table 2.** Results of the Bayesian Multilevel Random Model.

	n	Effect size	95% CI	Rhat
Overall	27	0.28	0.18 – 0.38	1
Peer Reviewed	13	0.34	0.23 – 0.46	1
Non Peer Reviewed	14	0.23	0.05 – 0.41	1

**Rhat =** ratio of the average variance of samples within each chain to the variance of the pooled samples across chains. CI – Credible Intervals.

Sensitivity analysis of the overall effect size, didn’t reveal any change from Rho 0 to Rho 1, suggesting that the degree of correlations among the dependent effect sizes don’t affect its magnitude.

Another “sensitivity analysis” was carried out excluding the new Mossbridge and Tressoldi studies in order to control whether different authors could obtain similar results. The main results of this analysis by using the same frequentist multilevel random model, is reported in Table 3.

**Table 3.** Results of the frequentist multilevel random model without Mossbridge's and Tressoldi's studies.

	n	Effect size	95% CI	*p*	I ^2^	τ ^2^
Overall	21	0.22	0.05 – 0.39	0.013	81.5	0.061

I ^2^ = percentage of variation across studies that due to heterogeneity; τ ^2^ = Tau ^2^, variance of the true effect sizes. CI – Confidence Interval.

Both the frequentist and the Bayesian analyses support the evidence of an overall main effect of approximately .28, and a small difference between the peer and non-peer reviewed studies. These findings will be commented further in the discussion of the comparison with Mossbridge *et al*.

### Preregistered vs No-preregistered studies

This distinction is relevant for assessing the impact of the so-called Questionable Research Practices and in particular p-hacking ( Head *et al*., 2015; John *et al*., 2012). Preregistered studies must describe all details on how the data will be analyzed before their collection, thus reducing the degree of freedom available during and after data collection. It can be seen that preregistration makes a range of analytically spurious practices far less likely: from changing the type of data to be analysed, swapping secondary and primary hypotheses and creating new hypotheses post hoc and other practices aimed at artificially inflating the “true” effect size.

From our database it was possible to compare the estimate of the effect size obtained from the pre-registered studies with that obtained from the no-preregistered ones. The results are presented in the following Table 4.

**Table 4.** Preregistered vs No-preregistered effect size estimates.

	n	Effect size	95% CI	*p*	I ^2^	τ ^2^
Preregistered	14	0.31	0.18 – 0.45	4.3×10 ^-4^	79.4	0.035
Non- Preregistered	22	0.24	0.08 – 0.41	7.05×10 ^-3^	82.5	0.067

The effect size point estimates clearly show that the effect size of the preregistered studies is larger than that of the no-preregistered studies, however their precision estimates (see the 95% CI) reveal a considerable overlap and consequently they cannot be considered statistically different.

### Publication bias

Our very comprehensive literature search is likely to have reduced the probability of a publication bias. Nevertheless we added a statistical estimation of the publication bias.

Unfortunately, there is no consensus about what tests are statistically more valid ( Carter *et al*., 2017).

All the traditional tests, like the Fail-Safe, the Trim-and-Fill, the Funnel Plot have been criticized for their limitations ( Jin *et al*., 2015; Rothstein, 2008). Similarly, more recent publication bias tests like the three-parameters selection model, the p-uniform* and the Vevea and Hedges’ weight-function model ( Vevea & Woods (2005), seem not recommended for multilevel random meta-analyses with high heterogeneity like the present one. Anyway, we applied the Copas selection model which is recommended by Jin *et al*. (2015). The Copas selection model was implemented using the metasens package ( Schwarzer *et al*., 2016), The results are presented in the Table 5. With this statistic, it emerges that there is no apparent statistical publication bias.

**Table 5.** Estimated effect size and corresponding 95% Confidence Intervals (CI) of the Copas Model.

	Effect size	95% CI
Copas Model adjusted	0.28	0.20 – 0.36

## Discussion

This update of the Mossbridge *et al.* (2012) meta-analysis related to the so called predictive anticipatory activity (PAA) responses to future random stimuli, covers the period January 2008- July 2018. Overall, we found 19 new studies describing a total of 36 effect sizes. Differently from the statistical approach of Mossbridge *et al*., in this meta-analysis we used a frequentist and a Bayesian multilevel model which allows an analysis of all effect sizes reported within a single study instead of averaging them.

Both the frequentist and the Bayesian analyses converged on similar results, making our findings quite robust. The overall effect size 0.28, 95% CI = 0.18 - 0.38, overlaps to that reported in the original paper: 0.21, 95% CI = 0.13–0.29, even if the heterogeneity is substantially higher: I ^2^= 81.9 vs 27.4.

The high level of heterogeneity is expected considering the varieties of experimental protocols and the diversity of dependent variables, from heart rate to pupil dilation.

Furthermore, we did not find substantial differences between peer and non-peer reviewed papers as in the original paper, as the confidence intervals of their mean effect size, overlap considerably.

## Conclusion

This update confirms the main results reported in Mossbridge *et al*. (2012) original meta-analysis and gives further support to the hypothesis of predictive physiological anticipatory activity of future random events. This phenomenon may hence be considered among the more reliable within those covered under the umbrella term “psi” (see Cardeña, 2018 for an exhaustive review of the evidence and the theoretical hypotheses of all these phenomena).

The limitations of the present meta-analysis are similar to most meta-analyses which include non-preregistered studies. The solution is that of prospective meta-analyses ( Watt & Kennedy, 2017), based on all preregistered studies where the methods and data analyses have been declared and made public beforehand.

As to the future of this line of research we think the time is now ripe for testing potential practical applications as suggested for example by Mossbridge *et al*. (2014). Franklin *et al*. (2014) and Khoshnoud *et al*. (2015).

In order to arrive at such an ambitious goal, it is necessary to achieve a high degree of correct classifications based on prestimulus activity at the level of each trial so that the number of false positives and false negatives is reduced to a bare minimum. The experiments of Mossbridge (2017); Baumgart *et al*. (2017) and Jolij & Bierman (2017) are promising examples in this regard.

## Data availability

The data referenced by this article are under copyright with the following copyright statement: Copyright: © 2018 Duggan M and Tressoldi P

Data associated with the article are available under the terms of the Creative Commons Zero "No rights reserved" data waiver (CC0 1.0 Public domain dedication). 

Underlying data for this meta-analysis is available from FigShare: https://doi.org/10.6084/m9.figshare.5661070.v3 ( Tressoldi, 2018) under a CC BY 4.0 licence

## Supplementary material

Supplementary File 1 – Completed PRISMA checklist.

Supplementary File 2 – List of references used in this analysis.

Supplementary File 3 – contains Table S1: Summary of the selected studies along with their corresponding effect sizes, variance and standard error. Table S2: results obtained with the multilevel approach suggested by Assink & Wibbelink, 2016.
