# Supplementary material for: Predictive physiological anticipatory activity preceding seemingly unpredictable stimuli: An update of Mossbridge et al’s meta-analysis
Source: F1000Res. 2018 Jul 17;7:407. Originally published 2018 Mar 28. [Version 2] doi: 10.12688/f1000research.14330.2 (PMC6124390; doi:10.12688/f1000research.14330.2)
Supplement: Supplementary file 1 [file f1000research-7-17025-s0000.tgz › 2a091ee2-9788-48cd-a098-e58a9ba9765c.docx]

| **Section/topic** | **#** | **Checklist item** | **Reported on page #** |
| --- | --- | --- | --- |
| **TITLE** | | |  |
| Title | 1 | meta-analysis |  |
| **ABSTRACT** | | |  |
| Structured summary | 2 | Background and Objective: This is an update of the Mossbridge et al’s meta-analysis related to the physiological anticipation preceding seemingly unpredictable stimuli.  Data source: Eighteen new peer and non peer reviewed studies were retrieved describing a total of 26 experiments and 34 associated effect sizes.  Study eligibility: Studies reporting psychophysiological measures before the presentation of random stimuli  Results: The overall weighted effect size, estimated with a frequentist multilevel random model, was: .29; 95% Confidence Intervals: .19 - .38; The overall weighted effect size, estimated with a multilevel Bayesian model, was: .29; 95% Credible Intervals: .18 - .39.  Effect sizes of peer reviewed studies were slightly higher: .38; Confidence Intervals: .27 - .48than not peer reviewed ones: .22; Confidence Intervals: .05 - .39.  Publication Bias: The statistical estimation of the publication bias by using the Copas model suggest that the main findings are not contaminated by publication bias.  Conclusions: In summary, with this update, the main findings reported in Mossbridge et al’s meta-analysis, are confirmed. |  |
| **INTRODUCTION** | | |  |
| Rationale | Pag. 3 | Described the rationale for the review in the context of what is already known. |  |
| Objectives | Pag. 4 | Provided an explicit statement of questions being addressed with reference to participants, interventions, comparisons, outcomes, and study design (PICOS). |  |
| **METHODS** | | |  |
| Protocol and registration | 5 | No review protocol |  |
| Eligibility criteria | Pag. 4 | Specified study characteristics (e.g., PICOS, length of follow-up) and report characteristics (e.g., years considered, language, publication status) used as criteria for eligibility, giving rationale. |  |
| Information sources | Pag. 4 | Described all information sources (e.g., databases with dates of coverage, contact with study authors to identify additional studies) in the search and date last searched. |  |
| Search | Pag. 4 | Presented full electronic search strategy for at least one database, including any limits used, such that it could be repeated. |  |
| Study selection | Pag. 5 | Stated the process for selecting studies (i.e., screening, eligibility, included in systematic review, and, if applicable, included in the meta-analysis). |  |
| Data collection process | Pag. 5 | Described method of data extraction from reports (e.g., piloted forms, independently, in duplicate) and any processes for obtaining and confirming data from investigators. |  |
| Data items | Pag. 6 | Listed and defined all variables for which data were sought (e.g., PICOS, funding sources) and any assumptions and simplifications made. |  |
| Risk of bias in individual studies | Pag. 8 | Described methods used for assessing risk of bias of individual studies (including specification of whether this was done at the study or outcome level), and how this information is to be used in any data synthesis. |  |
| Summary measures | Pag. 8 | Stated the principal summary measures: Hedges’ g |  |
| Synthesis of results | Pag. 8 | Described the methods of handling data and combining results of studies, if done, including measures of consistency (e.g., I^2^) for each meta-analysis. |  |

Page 1 of 2

| **Section/topic** | **#** | **Checklist item** | **Reported on page #** |
| --- | --- | --- | --- |
| Risk of bias across studies | Pag.  8-9 | Publication bias was analysed by using the Copas method |  |
| Additional analyses | Pag. 8 | subgroup analyses: Peer vs No Peer-reviewer papers |  |
| **RESULTS** | | |  |
| Study selection | 17 | flow diagram on pag, 6 |  |
| Study characteristics | 18 | Database and all included studies, available open access on https://doi.org/10.6084/m9.figshare.5661070.v1 |  |
| Risk of bias within studies | 19 | NA |  |
| Results of individual studies | 20 | forest plot on pag. 7 |  |
| Synthesis of results | 21 | See Tables: 1,2,3 a Supplementary analysis |  |
| Risk of bias across studies | 22 | See Table 4 |  |
| Additional analysis | 23 | See tables 2, 3 and Supplemental Analysis |  |
| **DISCUSSION** | | |  |
| Summary of evidence | Pag. 9 | Summarized the main findings including the strength of evidence for each main outcome; consider their relevance to key groups (e.g., healthcare providers, users, and policy makers). |  |
| Limitations | Pag. 10 | Discussed limitations at study and outcome level (e.g., risk of bias), and at review-level (e.g., incomplete retrieval of identified research, reporting bias). |  |
| Conclusions | Pag. 9 | Provided a general interpretation of the results in the context of other evidence, and implications for future research. |  |
| **FUNDING** | | |  |
| Funding | 27 | No funding were available |  |

*From:*  Moher D, Liberati A, Tetzlaff J, Altman DG, The PRISMA Group (2009). Preferred Reporting Items for Systematic Reviews and Meta-Analyses: The PRISMA Statement. PLoS Med 6(6): e1000097. doi:10.1371/journal.pmed1000097

For more information, visit: **www.prisma-statement.org**.

Page 2 of 2
