# Supplementary material for: Predictive physiological anticipatory activity preceding seemingly unpredictable stimuli: An update of Mossbridge et al’s meta-analysis
Source: F1000Res. 2018 Jul 17;7:407. Originally published 2018 Mar 28. [Version 2] doi: 10.12688/f1000research.14330.2 (PMC6124390; doi:10.12688/f1000research.14330.2)
Supplement: Supplementary file 2 [file f1000research-7-17025-s0001.tgz › 926cb47e-2b82-4a61-8301-05f267b8b8a8.docx]

**References of the studies included in the meta-analysis**

D’León R. and Izara, N. (2018). Development of a Predictive Anticipatory Activity (PAA)

Software: A First Step towards a Medium-Term Goal. Proceedings of The Parapsychological Association Convention 2018, Palo Alto, CA

Duma, G. M., Mento, G., Manari, T., Martinelli, M., &Tressoldi, P. E. (2017). Driving with Intuition: A Preregistered Study About the EEG Anticipation of Simulated Random Car Accidents. *PLoSOne* 12(1): e0170370. doi:10.1371/journal.pone.0170370

Jolii, J. and Bierman, D. (2017a). Testing the Potential Paradoxes in “Retrocausal” Phenomena. In Quantum Retrocausation III, AIP Conf. Proc. 1841, 030002-1–030002-9; doi: 10.1063/1.4982774

Jolii, J. and Bierman, D. (2017b). Implicit psi in a stimulus detection task. Paper presented at the PA 60th Annual Convention. Athens.

McCraty, R., & Atkinson, M. (2014). Electrophysiology of intuition: pre-stimulus responses in group and individual participants using a Roulette paradigm. *Global Advances in Health and Medicine,* 3(2), 16-27.

Kittenis, M. (2011). Anomalous anticipatory event-related EEG activity in a face recognition memory task. Paper presented at the Parapsychological Association Annual Convention, Curitiba,Brasil.

May, E. C., &Spottiswoode, S. J. P. (2014). Anomalous Anticipatory Effects in the Human Autonomic Nervous System. In Edwin C. May & Sonali Bhatt Marwaha (Eds). Anomalous Cognition: Remote Viewing Research and Theory, pp. 152-157.

Mossbridge, J. A. (2014). Single-trial presentiment experiment. KPU Registry ID N. 1005.

Mossbridge, J. A. (2015). Single-trial confirmatory presentiment experiment. KPU Registry ID N. 1018.

Mossbridge, J. A. (2017). Characteristic Alpha Reflects Predictive Anticipatory Activity (PAA) in an Auditory-Visual Task. In International Conference on Augmented Cognition (pp. 79-89). Springer, Cham.

Radin, D. I., Vieten, C., Michel, L., & Delorme, A. (2011). Electrocortical Activity Prior to Unpredictable Stimuli in Meditators and Nonmeditators. *Explore: The Journal of Science and Healing*, 7(5), 286-299.

Rezaei, S., Mirzaei, M., &Zali, M. R. (2014). Nonlocal intuition: replication and paired-subjects enhancement effects. *Global Advances in Health and Medicine*, 3(2), 5-15.

Savva, L. (2014). Is some of the evidence for ostensible precognition indicative of Darwinian adaptation to retrocausalinfluences?. Dissertation. Department of Psychology, Goldsmiths College. <http://research.gold.ac.uk/10721/>

Siller A, Ambach W and Vaitl D (2015). Investigating expectation effects using multiple physiological measures. *Frontiers in Psychology*,.6:1553. doi: 10.3389/fpsyg.2015.01553

Silva, F.E. (2015). El hipotéticoefecto de anticipación anómala de stímulos aparentemente impredecibles ¿podría afectar la toma de decision humana? In A. Parra (Ed). Ojos Invisibles. Editorial Antigua. Buenos Aires.

Singh, P.K. (2009). Personality correlates to electrophysiological measures of prestimulus response. Dissertation presented to Institute of Transpersonal Psychology,Palo Alto, California.

Tressoldi, P. E., Martinelli, M., Semenzato, L., & Cappato, S. (2011). Let your eyes predict: prediction accuracy of pupillary responses to random alerting and neutral sounds. *Sage Open*, *1*(2), doi.org/10.1177/2158244011420451

Tressoldi, P. E., Martinelli, M., & Semenzato, L. (2014). Pupil dilation prediction of random events. *F1000Research*, 2:262; doi: 10.12688/f1000research.2-262.v2.

Tressoldi, P. E., Martinelli, M., Semenzato, L., & Gonella, A. (2015). Does psychophysiological predictive anticipatory activity predict real or future probable events? *EXPLORE: The Journal of Science and Healing*, 11(2), 109-117.
