# Supplementary material for: Predictive physiological anticipatory activity preceding seemingly unpredictable stimuli: An update of Mossbridge et al’s meta-analysis
Source: F1000Res. 2018 Jul 17;7:407. Originally published 2018 Mar 28. [Version 2] doi: 10.12688/f1000research.14330.2 (PMC6124390; doi:10.12688/f1000research.14330.2)
Supplement: Supplementary file 3 [file f1000research-7-17025-s0002.tgz › 8a448822-8c4b-4905-bb73-06e7f40746c9.docx]

**Table S1:**

| **Author** | **Year** | **ExpID** | **nTot** | **randomisation** | **PeerRev** | ***g*** | **vg** | **se** | **esID** |
| --- | --- | --- | --- | --- | --- | --- | --- | --- | --- |
| **Duma et al.** | 2017 | 1 | 40 | pseudo | **yes** | 0.255 | 0.025 | 0.158 | 1 |
|  |  | 1 | 40 | pseudo | yes | 0.275 | 0.025 | 0.158 | 2 |
| D’León & Izara | 2018 | 2 | 39 | true | **yes** | 0.228 | 0.025 | 0.159 | 3 |
|  |  | 2 | 39 | true | **yes** | 0.091 | 0.025 | 0.157 | 4 |
| Jolii & Bierman | 2017 | 3 | 102 | true | **no** | 0.635 | 0.012 | 0.108 | 5 |
| Jolii & Bierman | 2017 | 4 | 105 | true | **no** | 0.196 | 0.010 | 0.098 | 6 |
| Kittenis | 2011 | 5 | 20 | pseudo | **no** | 0.475 | 0.052 | 0.227 | 7 |
| May & Spoottiswoode | 2014 | 6 | 100 | true | **no** | 0.504 | 0.011 | 0.105 | 8 |
| **McCraty** | 2014 | 7 | 13 | true | **yes** | 0.332 | 0.072 | 0.268 | 9 |
|  |  | 7 | 13 | true | yes | 0.434 | 0.075 | 0.273 | 10 |
| Mossbridge | 2017 | 8 | 40 | pseudo | **no** | 0.549 | 0.028 | 0.167 | 11 |
| Mossbridge | 2015 | 9 | 164 | pseudo | no | 0.105 | 0.006 | 0.078 | 12 |
|  |  | 10 | 164 | pseudo | no | 0.001 | 0.006 | 0.078 | 13 |
| Mossbridge | 2014 | 11 | 143 | pseudo | no | 0.137 | 0.007 | 0.084 | 14 |
|  |  | 12 | 145 | pseudo | no | 0.356 | 0.007 | 0.085 | 15 |
| **Radin et al.** | 2011 | 13 | 16 | true | **yes** | 0.104 | 0.057 | 0.238 | 16 |
| Rezaei | 2014 | 14 | 45 | pseudo | **yes** | 0.312 | 0.023 | 0.150 | 17 |
| Singh | 2009 | 15 | 21 | true | no | 0.386 | 0.048 | 0.218 | 18 |
|  |  | 16 | 19 | true | no | 0.018 | 0.048 | 0.220 | 19 |
| Savva exp1 | 2014 | 17 | 60 | pseudo | no | -0.513 | 0.018 | 0.136 | 20 |
| Savva exp2 | | 18 | 60 | pseudo | no | 0.415 | 0.018 | 0.133 | 21 |
| **Siller et al.** | 2015 | 19 | 37 | pseudo | **yes** | -0.009 | 0.026 | 0.161 | 22 |
|  |  | 19 | 38 | pseudo | yes | 0.010 | 0.025 | 0.159 | 23 |
|  |  | 19 | 35 | pseudo | yes | -0.007 | 0.027 | 0.165 | 24 |
|  |  | 19 | 31 | pseudo | yes | 0.068 | 0.031 | 0.175 | 25 |
| Silva | 2015 | 20 | 944 | true | no | 0.050 | 0.001 | 0.033 | 26 |
| Tressoldi | 2011 | 21 | 80 | pseudo | **yes** | 0.327 | 0.013 | 0.114 | 27 |
| Tressoldi exp1 | 2014 | 22 | 100 | pseudo | **yes** | 0.427 | 0.011 | 0.104 | 28 |
| Tressoldi exp2 | | 23 | 100 | pseudo | yes | 0.645 | 0.012 | 0.109 | 29 |
| Tressoldi exp1 | 2015 | 24 | 100 | pseudo | **yes** | 0.377 | 0.011 | 0.103 | 30 |
|  |  | 24 | 100 | pseudo | yes | 0.675 | 0.012 | 0.110 | 31 |
| Tressoldi exp2 | | 25 | 100 | pseudo | **yes** | 0.447 | 0.011 | 0.104 | 32 |
|  |  | 25 | 100 | pseudo | yes | 0.516 | 0.011 | 0.106 | 33 |
| Tressoldi exp3 | | 26 | 80 | pseudo | **yes** | 0.258 | 0.013 | 0.113 | 34 |
| Tressoldi exp4 | | 27 | 80 | pseudo | **yes** | 0.386 | 0.013 | 0.115 | 35 |
|  |  | 27 | 80 | pseudo | yes | 0.485 | 0.014 | 0.117 | 36 |

Table S2: Results of the frequentist random model following Assink et al. 2016 method

|  | n | Effect size | 95% Conf. Int. | *p* |
| --- | --- | --- | --- | --- |
| Overall | 27 | 0.28 | 0.18 - 0.37 | 2.6x10^-9^ |
| Peer Review | 13 | 0.34 | 0.24 - 0.44 | 2.06x10^-11^ |
| No Peer Review | 14 | 0.23 | 0.07 - 0.38 | .004 |
