# Supplementary material for: Predictive physiological anticipatory activity preceding seemingly unpredictable stimuli: An update of Mossbridge et al’s meta-analysis
Source: F1000Res. 2018 Jul 17;7:407. Originally published 2018 Mar 28. [Version 2] doi: 10.12688/f1000research.14330.2 (PMC6124390; doi:10.12688/f1000research.14330.2)
Supplement: Peer review history

# Peer review: Referee response for version 2

**DOI:** 10.5256/f1000research.17025.r36146

**Published:** 2018-09-04

**Authors:** David Vernon

**Affiliations:** 1 Canterbury Christ Church University (CCCU), Kent, UK

I'm happy that the authors have addressed most of my prior comments - however I would still liked to have seen a more fuller discussion of what these results entail and their implications.

I have read this submission. I believe that I have an appropriate level of expertise to confirm that it is of an acceptable scientific standard.

# Peer review: Referee response for version 2

**DOI:** 10.5256/f1000research.17025.r36147

**Published:** 2018-08-01

**Authors:** Stephen Baumgart

**Affiliations:** 1 Department of Psychology and Brain Sciences, University of California, Santa Barbara, Santa Barbara, CA, USA

I think the addition of the "Preregistered versus No-pregistered" section, analysis, and table adequately satisfies the serious concerns that I have about p-hacking. I switch my status to "Approved". Even though I'm still concerned about including non-preregistered studies at all, I realize that such a restriction does not have strong precedent. But I would still encourage authors to focus on meta-analysis of preregistered studies in the future.

 One minor comment which I have on the writing is that "No-pregistered" should be "Non-preregistered".

I have read this submission. I believe that I have an appropriate level of expertise to confirm that it is of an acceptable scientific standard.

# Peer review: Referee response for version 1

**DOI:** 10.5256/f1000research.15593.r35197

**Published:** 2018-07-05

**Authors:** Stephen Baumgart

**Affiliations:** 1 Department of Psychology and Brain Sciences, University of California, Santa Barbara, Santa Barbara, CA, USA

**Addressing Major Criticisms**

 This is a controversial topic and careful consideration of objections is needed in a meta-analysis. Presentiment or Predictive Physiological Anticipation Studies (PAA) are typically criticized on these grounds (see, for example, Wagenmakers, Wetzels, Borsboom, Kievit, van der Maas, 2015):

1. Physical impossibility
2. File-drawer effect
3. Biases due to multiple comparisons or p-hacking

As for the first criticism, discussion of physical plausibility is beyond the scope of this meta-analysis and is left to the discretion of the authors. Nevertheless, apparent violations of our intuitions of time are found at the quantum level, such as the Wheeler Delayed-Choice experiment. It is not impossible that such effects may scale up to a macroscopic level in a not-yet-understood emergent process. I think the final two sentences of the introduction satisfy considerations of the physical impossibility objection and no changes are needed.

 Though file-drawer effects are frequently cited as a serious concern, the results section adequately discusses this issue. However, expert review is needed for this area (my response to "Is the statistical analysis and its interpretation appropriate? " should really be a combination of "Partly" and "A qualified statistician is needed".) I agree with the first sentence of the "Publication Bias" subsection that publication bias is not that serious of a concern because of the limited number of researchers and available funding.

 By far the most serious concern is the third, that of multiple comparisons or p-hacking, which I do not believe is adequately addressed by either the discussion or conclusion sections. Two sentences in the conclusion are not sufficient to address this serious concern. I have included recommendations later in this review. I am aware the authors already know the following but by doing multiple analyses and only reporting a sub-sample of them a believer or supporter of a hypothesis could bias effect sizes up while a skeptic or opponent could bias effect sizes down (and none of these biases are necessarily intentional or even conscious).

 In the context of PAA, serious sources of p-hacking concern are establishing baselines for electrophysiological data, deciding time regions for analysis, and methodologies for rejecting bad data and artifacts. For some physiological measurements, the problem is even worse. In Electroencephalography (EEG) studies, for example, a researcher could either study event-related potentials (ERPs), the spectral power densities of various oscillations, or the phases of such oscillations, or a host of other possible analyses. Considering oscillations, the frequency range of an analysis can also be freely selected. Additionally, a researcher could select different bandpass filters to use or even which section of the head is included in the analysis. This is in addition to the concerns with artifact rejection, time region, and baselining already discussed. With so many free parameters, a non-preplanned study is practically useless as hard evidence for an effect unless the statistical significance of the effect is high enough that it becomes implausible that the effect in question can be generated by tweaking free parameters. Even if the statistical significance is high, the effect size is still untrustworthy because an analyst could be tweaking parameters in an effort to improve the analysis or fix problems but is only homing in on statistical fluctuations. These concerns are one reason why I refused to include exploratory EEG research from my own lab in this meta-analysis.

 The solution to the multiple analysis problem is to separate research into exploratory studies where adjustments can be made in analysis and pre-planned confirmatory studies. Some of the studies included in the meta-analysis are pre-planned confirmatory studies, which should be considered the only truly reliable results for estimates of effect size due to the concerns laid out in this review (even for confirmatory studies, mistakes by researchers could distort effect sizes but these mistakes may average out in the long run).

 My recommended solutions for this paper are:

1. More discussion of the risks of p-hacking in biasing results in the discussion section
2. Separated analyses of pre-registered confirmatory studies and exploratory studies and discussion comparing the two
3. For exploratory studies in the study tables, include the experimenter expectation of whether the hypothesis will be verified (such as in Galak, LeBoeuf, Nelson, & Simmons, 2012)
4. Show whether multiple comparison corrections were made for exploratory studies

Exploratory studies are necessary for advancing the field. But a meta-analysis should not include them without major caveats due to potential distortions of the effect size.

 I am aware the extra attention given to p-hacking risks in this research is not precedented by other fields but the small effect sizes and the major implications to our understanding of physics, psychology, and neuroscience PAA research engenders may justify additional caution be used. My colleagues and I discuss this further in Schooler, Baumgart, & Franklin, 2018.

**Other Comments**

1. “The presentiment hypothesis calls for a difference between arousing and neural pre-stimulus response and this is calculated across sessions” is not always true. For example, the hypothesis could also cover the difference between two different types of arousing stimulus (for example, auditory versus visual stimulus or two different types of visual stimulus).
2. Further discussion should be included for the observations mentioned of the second-to-last paragraph of the discussion; otherwise, it may be unclear why these studies are interesting as the paper asserts.

I have read this submission. I believe that I have an appropriate level of expertise to confirm that it is of an acceptable scientific standard, however I have significant reservations, as outlined above.

## References

[1] GalakJLeboeufRANelsonLDSimmonsJP: Correcting the past: failures to replicate ψ. *J Pers Soc Psychol*.2012;103(6) : 10.1037/a0029709 933-48 10.1037/a0029709 22924750

[2] SchoolerJBaumgartSFranklinM: Entertaining without endorsing: The case for the scientific investigation of anomalous cognition. *Psychology of Consciousness: Theory, Research, and Practice*.2018;5(1) : 10.1037/cns0000151 63-77 10.1037/cns0000151

[3] SimmonsJPNelsonLDSimonsohnU: False-positive psychology: undisclosed flexibility in data collection and analysis allows presenting anything as significant. *Psychol Sci*.2011;22(11) : 10.1177/0956797611417632 1359-66 10.1177/0956797611417632 22006061

[4] WagenmakersEJWetzelsRBorsboomDKievitRAvan der Maas.HLJ: A Skeptical Eye on Psi. In E. C. May and S. B. Marwaha (Eds.), Extrasensory Perception: Support, Skepticism, and Science. *Santa Barbara,CA: ABC-CLIO*.2015;285-316

# Response

**Published:** 2018-07-12

**Authors:** Patrizio Tressoldi

**Affiliations:** Dipartimento di Psicologia Generale, Università di Padova, Italy

Thank you for your detailed and constructive comments.

 Here it follows our replies to your main comments.

 Though file-drawer effects are frequently cited as a serious concern, the results section adequately discusses this issue. However, expert review is needed for this area (my response to "Is the statistical analysis and its interpretation appropriate? " should really be a combination of "Partly" and "A qualified statistician is needed".) I agree with the first sentence of the "Publication Bias" subsection that publication bias is not that serious of a concern because of the limited number of researchers and available funding.

**Reply: we think we have a sufficient expertise in dealing with this problem. Furthermore we consulted with R.C.M. van Aert who is an expert on this topic.**

 My recommended solutions for this paper are:

1. More discussion of the risks of p-hacking in biasing results in the discussion section
2. Separated analyses of pre-registered confirmatory studies and exploratory studies and discussion comparing the two

**Reply: we have added a direct comparison between preregistered and no-preregistered studies, see Table 4 and the paragraph “Preregistered vs No-preregistered studies”**


**  **

1. For exploratory studies in the study tables, include the experimenter expectation of whether the hypothesis will be verified (such as in Galak, LeBoeuf, Nelson, & Simmons, 2012)

**Reply: Unfortunately no one study checked this moderating variable, but our sensitivity analysis reported in Table 3, suggests that the experimenter expectation did not affect considerably the overall results.**


**  **

1. Show whether multiple comparison corrections were made for exploratory studies

**Reply: our choice to use multivariate analyses, partly reduce the impact of this procedure.**

  

1. “The presentiment hypothesis calls for a difference between arousing and neural pre-stimulus response and this is calculated across sessions” is not always true. For example, the hypothesis could also cover the difference between two different types of arousing stimulus (for example, auditory versus visual stimulus or two different types of visual stimulus).

**Reply: revised as “The presentiment hypothesis calls for a difference between the pre-stimulus responses of the two stimulus categories..”**


**  **

1. Further discussion should be included for the observations mentioned of the second-to-last paragraph of the discussion; otherwise, it may be unclear why these studies are interesting as the paper asserts.

**Reply: we expanded our conclusion as suggested.**

# Peer review: Referee response for version 1

**DOI:** 10.5256/f1000research.15593.r32577

**Published:** 2018-04-10

**Authors:** David Vernon

**Affiliations:** 1 Canterbury Christ Church University (CCCU), Kent, UK

**Introduction**

 P.2, Line 21: Not sure I would agree with ‘body predicting moments ahead of time’ as this suggests understanding – try ‘reacting ahead of time’ or simply ‘physiological changes ahead…’

 P.2: Para 2: the authors note that two paradigms were used, presentation of arousing/neutral stimuli or guessing tasks. Were any clear differences in PAA effects reported between these tasks?

 Also, given the ‘broad range of physiological measures’ used to assess such changes were there any key differences here?

 P.2, Para 2, final sentence: the ‘evidence from mainstream research’ – what specifically does this refer to? Behavioural effects? Ie changes in accuracy and/or response times and if so could do with a clear reference.

 P. 2, Para 3, line 9: ‘forwarded’ doesn’t make sense. Do you mean ‘proposed as a potential framework/theory’?

 P. 2, Para 3: Not sure I’d agree that using physiological markers to ‘predict’ future events is a ‘second objective’ measure. It is simply another way to view the same procedure.

 P. 2, Para 3: the vague references to ‘presentiment piggybacking onto mainstream research’ needs clarifying and supporting with references.

**Methods**

 P.2, Para 1: need to identify the acronym ‘PRISMA’ after it is outlined.

 P. 2, Para 3, line 3: change ‘were’ to ‘where’

 ……………….., line 4: change ‘Differently’ to ‘In addition,’

 Also, what is the rationale for utilising a distinct eligibility criterion? It seems that prior research focused on testing for a pre-stim signal that would match the post-stim presentation. By not using this method you open yourself up to the criticism of widening the scope and also of looking for ‘any physiological change’ as opposed to one that would be specifically linked to the presentation of the target. The authors claim this is ‘more comprehensive’ but it could just as easily be seen as less conservative.

 P.3, line 4: change to ‘this type of investigation’

 Line 8: change ‘investigations’ to ‘research’.

 Line 8: The point about studies possibly ‘missed’ by Mossbridge et al is not clear. What makes you think any studies were ‘missed’ and why did you then include the same time period – ie from 2008 to 2010 – if you are ‘adding’ to the data it would make sense to begin your inclusion time from 2010 unless you have evidence that some studies were ‘missed’?

 P.3, Para 4: line 1: change ‘were studies were’ to ‘were studies where’

 P.3 – is it possible to say a bit more about why some authors did not agree to share their data – looks distinctly odd.

 P.4, Para 6: sentence referring to ‘Assink’ doesn’t make sense – unless you move the ref out of parenthesis and into the sentence.

 P.4: Change ‘The Bayesian’ to ‘A Bayesian’. And pull the sentence with syntax to the same paragraph.

 P. 4: Change: ‘Even if with our search activity we are quite….’ To ‘The robust search is likely to have reduced the probability of a publication bias occurring. Nevertheless, to test this a statistical estimation was conducted using the Copas selection model, as recommended by Jin et al’

**Results**

 Keep tense to past ie peer reviewed not review.

 It doesn’t make sense to compare data from the current review to Mossbridge et al ‘if’ both sets of data contain the same studies – as this would lead to obvious similarities etc. To an extent this seems to be addressed by the data in Table 3 but not made clearly – ie why not simply state that when X studies were excluded due to Y reasons the overall effect was still significant?

 I don’t see the moderation results for PeerRev reported here?

 The reported ‘small difference between the peer reviewed and non-peer reviewed’ is vague and unhelpful. State clearly what was found – ie, are they ‘significantly different’ if not then they are not ‘different’ in any meaningful way.

 Under ‘Publication bias’ I think para 2, 3 and 4 (which appears on P.6) should be joined as one single paragraph.

**Discussion**

 This is rather poor and reads like a list of points. There needs to be some discussion here not simply a repetition of the data. Ie – given this effect size how would the authors attempt to account for it? what are the implications of such a finding? Is there any scope for teasing out of the data any factors that may/may not influence the outcome – e.g., a possible relationship between the PAA and the various DV measures used?

 The point relating to the work of Jolij and Bierman is again vague and unclear. What evidence precisely are you referring to here and how/why is this similar to the ‘psychological research’ [and what does this refer to?]

 What is the ‘conventional research program’ of Kittenis?

 How, exactly, does the single trial work of Mossbridge counter QRP?

I have read this submission. I believe that I have an appropriate level of expertise to confirm that it is of an acceptable scientific standard, however I have significant reservations, as outlined above.

# Response

**Published:** 2018-07-12

**Authors:** Patrizio Tressoldi

**Affiliations:** Dipartimento di Psicologia Generale, Università di Padova, Italy

Thank you for your detailed and constructive comments.

 Here it follows our replies to your main comments.

**Introduction**

 P.2, Line 21: Not sure I would agree with ‘body predicting moments ahead of time’ as this suggests understanding – try ‘reacting ahead of time’ or simply ‘physiological changes ahead…’

 P.2, Line 21: Not sure I would agree with ‘body predicting moments ahead of time’ as this suggests understanding – try ‘reacting ahead of time’ or simply ‘physiological changes ahead…’

**Reply: we changed with “‘physiological changes ahead of time”.**

 P.2: Para 2: the authors note that two paradigms were used, presentation of arousing/neutral stimuli or guessing tasks. Were any clear differences in PAA effects reported between these tasks?

**Reply: No**

 Also, given the ‘broad range of physiological measures’ used to assess such changes were there any key differences here?

**Reply: No**

 P.2, Para 2, final sentence: the ‘evidence from mainstream research’ – what specifically does this refer to? Behavioural effects? Ie changes in accuracy and/or response times and if so could do with a clear reference.

**Reply: Added reference**

 P. 2, Para 3, line 9: ‘forwarded’ doesn’t make sense. Do you mean ‘proposed as a potential framework/theory’?

**Reply: replaced with "proposed as a potential mechanism".**

 P. 2, Para 3: Not sure I’d agree that using physiological markers to ‘predict’ future events is a ‘second objective’ measure. It is simply another way to view the same procedure.

**Reply: changed as “another way..”**

 P. 2, Para 3: the vague references to ‘presentiment piggybacking onto mainstream research’ needs clarifying and supporting with references.

**Reply: deleted this paragraph**

**Methods**

 P.2, Para 1: need to identify the acronym ‘PRISMA’ after it is outlined.

**Reply: added**

 P. 2, Para 3, line 3: change ‘were’ to ‘where’

 ……………….., line 4: change ‘Differently’ to ‘In addition,’

**Reply: changed accordingly**

 Also, what is the rationale for utilising a distinct eligibility criterion? It seems that prior research focused on testing for a pre-stim signal that would match the post-stim presentation. By not using this method you open yourself up to the criticism of widening the scope and also of looking for ‘any physiological change’ as opposed to one that would be specifically linked to the presentation of the target. The authors claim this is ‘more comprehensive’ but it could just as easily be seen as less conservative.

**Reply: We prefer the term more comprehensive because some experimental designs, e.g. hit guessing, don’t allow a post-stimulus physiological measure. However, all experimental designs tied the differential anticipatory physiological activity to two different outcomes, e.g. hits or misses.** 

 P.3, line 4: change to ‘this type of investigation’

 Line 8: change ‘investigations’ to ‘research’.

**Reply: fixed.**

 Line 8: The point about studies possibly ‘missed’ by Mossbridge et al is not clear. What makes you think any studies were ‘missed’ and why did you then include the same time period – ie from 2008 to 2010 – if you are ‘adding’ to the data it would make sense to begin your inclusion time from 2010 unless you have evidence that some studies were ‘missed’?

**Reply: after Mossbridge et al publication, we discovered that Singh, P.K. (2009), was missed.**

 P.3, Para 4: line 1: change ‘were studies were’ to ‘were studies where’

**Reply: fixed.**

 P.3 – is it possible to say a bit more about why some authors did not agree to share their data – looks distinctly odd.

**Reply:** **the reasons for such decisions are confidential.**

 P.4, Para 6: sentence referring to ‘Assink’ doesn’t make sense – unless you move the ref out of parenthesis and into the sentence.

**Reply: fixed**

 P.4: Change ‘The Bayesian’ to ‘A Bayesian’. And pull the sentence with syntax to the same paragraph.

**Reply: fixed**

 P. 4: Change: ‘Even if with our search activity we are quite….’ To ‘The robust search is likely to have reduced the probability of a publication bias occurring. Nevertheless, to test this a statistical estimation was conducted using the Copas selection model, as recommended by Jin et al’

**Reply: fixed**

**Results**

 Keep tense to past ie peer reviewed not review.

**Reply: fixed**

 It doesn’t make sense to compare data from the current review to Mossbridge et al ‘if’ both sets of data contain the same studies – as this would lead to obvious similarities etc. To an extent this seems to be addressed by the data in Table 3 but not made clearly – ie why not simply state that when X studies were excluded due to Y reasons the overall effect was still significant?

**Reply: we clarified that the Mossbridge and Tressoldi's studies were those included in this update.**

 I don’t see the moderation results for PeerRev reported here?

**Reply:  we think this analysis redundant given the data reported on** **Tables 1 and 2**

 The reported ‘small difference between the peer reviewed and non-peer reviewed’ is vague and unhelpful. State clearly what was found – ie, are they ‘significantly different’ if not then they are not ‘different’ in any meaningful way.

**Reply: we clarified that the means are different, but their precision estimate, i.e. confidence intervals, overlap.**

 Under ‘Publication bias’ I think para 2, 3 and 4 (which appears on P.6) should be joined as one single paragraph.

**Reply: fixed**

**Discussion**

 This is rather poor and reads like a list of points. There needs to be some discussion here not simply a repetition of the data. Ie – given this effect size how would the authors attempt to account for it? what are the implications of such a finding? Is there any scope for teasing out of the data any factors that may/may not influence the outcome – e.g., a possible relationship between the PAA and the various DV measures used?

**Reply: we changed the discussion and the conclusion to include our evaluation of the status of art and the future of this phenomenon.**

 What is the ‘conventional research program’ of Kittenis?

**Reply:** **omitted**

 How, exactly, does the single trial work of Mossbridge counter QRP?

**Reply: we wrote “pre-registered single-trial work”. Preregistration of data analyses constraints the use of post-hoc data analysis flexibility.**
